# Artificial Intelligence-Assisted Infrared Spectroscopy and Chemometrics for Enhanced Histopathology Screening of Micro- and Macrocancer Lesions

**DOI:** 10.1021/acs.analchem.5c06870

**Published:** 2026-02-02

**Authors:** Karolina Chrabaszcz, Guillermo Quintas, Julia Kuligowski, Kamilla Malek

**Affiliations:** 1 Institute of Nuclear Physics, Polish Academy of Sciences, Radzikowskiego 152, Krakow 31-342, Poland; 2 Leitat Technological Center, Avenida Fernando Abril Martorell, Torre 106 A, Valencia 46026, Spain; 3 Neonatal Research Group, Health Research Institute La Fe, Avenida Fernando Abril Martorell, Torre 106 A, Valencia 46026, Spain; 4 Spanish Network in Maternal, Neonatal, Child and Developmental Health Research (RICORS-SAMID) (RD24/0013/0014), Instituto de Salud Carlos III, Madrid 28029, Spain; 5 Servicio de Análisis de Vesículas Extracelulares (SAVE), Health Research Institute Hospital La Fe (IIS La Fe), Avda. Fernando Abril Martorell 106, Valencia 46026, Spain; 6 Faculty of Chemistry, Jagiellonian University in Krakow, Gronostajowa 2, Krakow 30-38, Poland

## Abstract

Accurate detection of micro- and macrocancer lesions remains a critical challenge in histopathology, as conventional hematoxylin and eosin staining requires labor-intensive analysis and is limited in sensitivity toward microscopic foci. Here, we present an artificial intelligence (AI)-assisted workflow integrating Fourier transform infrared (FT-IR) hyperspectral imaging with chemometric modeling for enhanced cancer screening in lung tissues. Using a focal-plane array (128 × 128 pixels with a pixel projection of 5.5 μm × 5.5 μm), hyperspectral maps were generated, enabling biochemical characterization of distinct morphological structures, including bronchial and vascular walls, parenchyma, and neoplastic regions. Histopathological annotations were employed to construct calibration data sets for noncancerous tissues, microcancer lesions, and macrocancer lesions. Discriminant analysis revealed high predictive accuracy across validation strategies, with CORRS-CV (δ = 5) outperforming conventional *k*-fold and image-based approaches (AUROC = 0.94, accuracy = 97%, and specificity = 98%). This robust performance reflects reduced cross-validation bias and improved generalizability of predictive models. Importantly, FT-IR imaging enabled the detection of both macro- and microlesions consistent with histological references, while also revealing spectral similarities in vascular walls that occasionally led to false-positive predictions. Together, these findings demonstrated that AI-assisted FT-IR chemometrics offers the rapid, label-free, and spatially resolved detection of cancer lesions, complementing standard histopathology by improving sensitivity to microscopic disease and supporting stratification of tumor progression.

## Graphical abstract

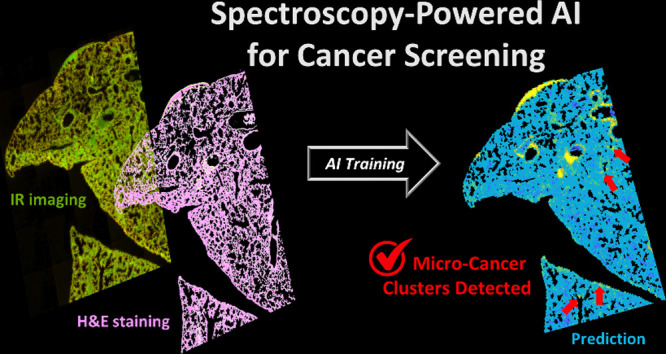

## Introduction

Histopathology, the microscopic examination of stained tissue sections, is the gold standard for clinical diagnosis, particularly in oncology, where it enables definitive tumor classification, grading, and staging.^1^ It provides unique insights into the tissue architecture and cellular morphology that remain indispensable despite advances in molecular and imaging-based diagnostics. However, the process is inherently labor-intensive and time-consuming, involving fixation, embedding, sectioning, staining, and manual assessment. Furthermore, interpretation is subject to interobserver variability, as diagnostic conclusions may differ among pathologists, especially for borderline or heterogeneous lesions. Increasing case volumes worldwide further exacerbate these challenges, contributing to extended turnaround times and workload pressures. These limitations underscore the growing need for standardization, digitization, and the integration of AI-assisted analytical methods, which hold promise for improving diagnostic accuracy, reproducibility, and efficiency in routine histopathological practice.^2,3^

While considerable pathological alterations or macroscopic lesions are relatively easy for a pathologist to identify, the real challenge lies in systematically scanning extensive tissue sections for subtle abnormalities. A standard core needle biopsy typically measures only 10–20 mm in length and 1–2 mm in diameter, which may seem small macroscopically; however, at ca. 50× magnification, this corresponds to a 10–40 mm^2^ tissue that must be manually and visually screened.^4,5^ Such screening can span multiple sessions across days, especially when searching for small tumor foci or even isolated malignant cells. In this context, integrating machine-learning methods capable of recognizing spectral or morphological signatures of malignant transformation offers a promising strategy: algorithms can prescreen slides and highlight regions of interest for subsequent expert verification, thereby improving sensitivity, reproducibility, and overall diagnostic efficiency.

Spectral histopathology imaging (SHI), an emerging discipline at the interface of spectroscopy and pathology, aims to complement or even partially replace conventional staining-based histology by providing label-free molecular fingerprints of cells and tissues.^6^ Among the most widely applied techniques is infrared (IR) spectroscopy, which enables nondestructive, rapid, and highly sensitive characterization of the biochemical composition of cells and extracellular matrix (ECM) components.^7,8^ In contrast to standard H&E staining, IR spectroscopy directly probes the vibrational modes of biomolecules such as proteins, lipids, nucleic acids, and carbohydrates, thereby enabling the identification of molecular alterations associated with tumorigenesis, progression, or therapeutic response. In histopathological applications, IR spectral imaging has been successfully used to discriminate between benign and malignant tissues, identify tumor margins, and even detect early neoplastic lesions invisible under light microscopy.^9–12^ Our previous studies on pulmonary lesions demonstrated that FT-IR spectral features directly reflect the biochemical and structural alterations associated with micro- and macrometastatic progression.^8,13^ Changes in the amide I (∼1685–1620 cm^–1^) and amide II (∼1540–1550 cm^–1^) regions with an elevated β-sheet content (∼1630–1640 cm^–1^) and increased cellular density were associated with tumor infiltration. In parallel, ECM remodeling accompanying metastatic advancement is evidenced by intensity variations and spectral shifts in collagen-associated bands within the 1200–1300 cm^–1^ range, including the emergence of features around ∼1297 cm^–1^. Collectively, these vibrational markers were consistently correlated with histopathological annotations of micro- and macrometastases in lung tissues. Importantly, lung cancer lesions frequently infiltrate vascular and bronchiolar structures, leading to partial overlap between cancer-related FT-IR signatures and biochemical features characteristic of collagen-rich vessel walls and remodeled ECM. In particular, overlapped shared vibrational contributions in the fingerprint region (1200–1000 cm^–1^), including bands around 1200–1300 and ∼1080 cm^–1^, can complicate automated discrimination between neoplastic infiltration and nonmalignant vascular structures.

Recent advances in FT-IR instrumentation and data acquisition have substantially expanded its diagnostic applicability. In particular, the introduction of focal-plane array (FPA) detectors, quantum cascade laser (QCL)-based infrared sources, and improved optical designs has enabled fast, high-throughput acquisition of large hyperspectral data sets with enhanced spatial resolution and molecular specificity.^13–16^ These technological developments enable analysis of entire tissue sections within clinically relevant timeframes, bridging the gap between spectroscopy and practical histopathological workflows. In parallel, the integration of FT-IR spectroscopy with chemometric and machine-learning approaches has significantly improved its diagnostic performance, enabling reliable discrimination between healthy, premalignant, and malignant samples across multiple cancer types.^17–19^ Attenuated total reflection FT-IR (ATR-FT-IR) has demonstrated strong potential for minimally invasive cancer diagnostics through the analysis of serum, plasma, and urine.^20–22^ Beyond liquid biopsies, FT-IR spectral imaging has been successfully applied to solid tissues and cellular models, enabling molecular phenotyping of tumors, including oral, gastrointestinal, prostate, and lung cancers.^23–25^ Collectively, these advances establish FT-IR spectroscopy as a mature and versatile analytical platform for cancer detection and screening, offering a unique combination of chemical specificity, speed, and compatibility with digital pathology workflows.

Coupled with modern chemometric and machine-learning approaches, FT-IR-based SHI offers significant potential for automated, reproducible, and high-throughput diagnostics, while reducing reliance on labor-intensive staining protocols.^11,14,15^ Chemometrics provides advanced multivariate statistical tools for preprocessing, dimensionality reduction, and feature selection, which are crucial for handling complex, high-dimensional IR data sets obtained from heterogeneous tumor tissues. When integrated with machine-learning algorithms, including supervised classifiers such as support vector machines and random forests, as well as unsupervised clustering methods, SHI can achieve automated tissue segmentation, detection of tumor margins, and recognition of molecular phenotypes at cellular resolution.^12,22,26^ This synergy between FT-IR spectroscopy and AI-driven chemometric modeling not only accelerates data interpretation but also enhances reproducibility, paving the way toward reliable clinical translation of SHI diagnostics. However, the development and validation of robust machine-learning models for FT-IR-based SHI are complicated by the intrinsic spatial correlations in hyperspectral images. Standard internal validation approaches, such as repeated *k*-fold cross-validation, often yield overly optimistic performance estimates due to spectral oversampling, where adjacent pixels carry redundant biochemical information. This can artificially inflate classification accuracy and compromise its validation and clinical translation. To address this, the constrained repeated random subsampling–cross-validation (CORRS-CV) algorithm^27^ was introduced as a spatially aware validation strategy that enforces a minimum distance between training and test pixels and between training pixels. By mitigating replicate bias and accounting for the lateral spatial resolution of IR microscopy, CORRS-CV provides more realistic estimates of classifier generalizability. In the context of SHI, this could facilitate the validation and translation of FT-IR-based diagnostic workflows.

In this work, we focus on the detection of micro- and macrolesions in lung tissues arising from mammary cancer metastasis, which serves as a biologically relevant model for training and validating classifiers capable of recognizing both extensive and subtle cancer-associated alterations. FT-IR spectral histopathology constitutes the core analytical methodology, providing spatially resolved biochemical contrast based on intrinsic molecular fingerprints of tissue components. Chemometric and artificial intelligence approaches are employed exclusively as advanced data analysis tools to enhance the interpretation of FT-IR hyperspectral images, enabling automated identification and differentiation of cancerous and noncancerous regions with high accuracy. By preserving a clear methodological separation between spectroscopy-derived biochemical information and AI-assisted classification, the proposed workflow remains firmly rooted in FT-IR-based histopathology, while improving objectivity, reproducibility, and sensitivity in histopathological screening. Ultimately, this strategy supports early lesion detection, reduces observer-dependent variability, and contributes to the development of complementary diagnostic tools for clinical oncology.

## Materials and Methods

### Animal Model and Histological Staining

Lung tissues were obtained from inbred BALB/cAnNCrl mice, both healthy controls and 3 weeks after orthotopic implantation of viable 4T1 breast cancer cells, which are known to generate micro- and macrometastases.^28,29^ Following excision, the lungs were rinsed with saline and fixed in 4% buffered formalin for 48 h. Paraffin-embedded sections were prepared from the central region of the left lung. Using the paraffin technique, 7 μm slices were cut on an Accu-Cut SRM 200 rotary microtome, mounted on CaF_2_ slides, and subsequently dewaxed before FT-IR imaging. This fixation procedure ensured optimal preservation of tissue morphology for histological evaluation by preventing autolysis and cross-linking protein amine groups. Although fixatives are known to introduce certain molecular alterations, these changes were minor compared to the biochemical differences associated with pathology and did not interfere with spectral discrimination.^30^ After FT-IR imaging, the same sections were stained with hematoxylin and eosin (H&E) to provide a reference histopathological evaluation. Digital images of H&E-stained slides were acquired using an Olympus BX53F bright-field microscope equipped with a DP74 digital camera. All procedures were performed in accordance with the Guide for the Care and Use of Laboratory Animals (NIH) and were approved by the local animal ethics committee (permit no. LKE140/2013).

### FT-IR Spectroscopy Imaging

FT-IR imaging was performed using an Agilent 670-IR spectrometer coupled to a 620-IR microscope equipped with a focal-plane-array (FPA) detector. The detector comprised 16,384 pixels organized in a 128 × 128 grid. Spectral images were collected in transmission mode. A Cassegrain objective with a numerical aperture of 0.62 and an effective pixel size of 5.5 μm × 5.5 μm was applied.

### Multivariate Image Discriminant Analysis

Images containing FT-IR spectra (Fourier transform infrared) in the 1800–900 cm^–1^ range were imported into the MATLAB workspace. Prior to chemometric analysis, all spectra were preprocessed using second-derivative transformation and mean centering in the 900–1900 cm^–1^ region. IR images included tissue areas identified by a pathologist as C (cancerous), NC (noncancerous), and unlabeled (Table 1). Partial least-squares (PLS) classification models were built using labeled pixels for the discrimination of noncancerous (i.e., NC) vs cancerous areas (i.e., micro- (MICL) and macrocancer lesions (MACL)), Figure 1.

**Figure 1:**
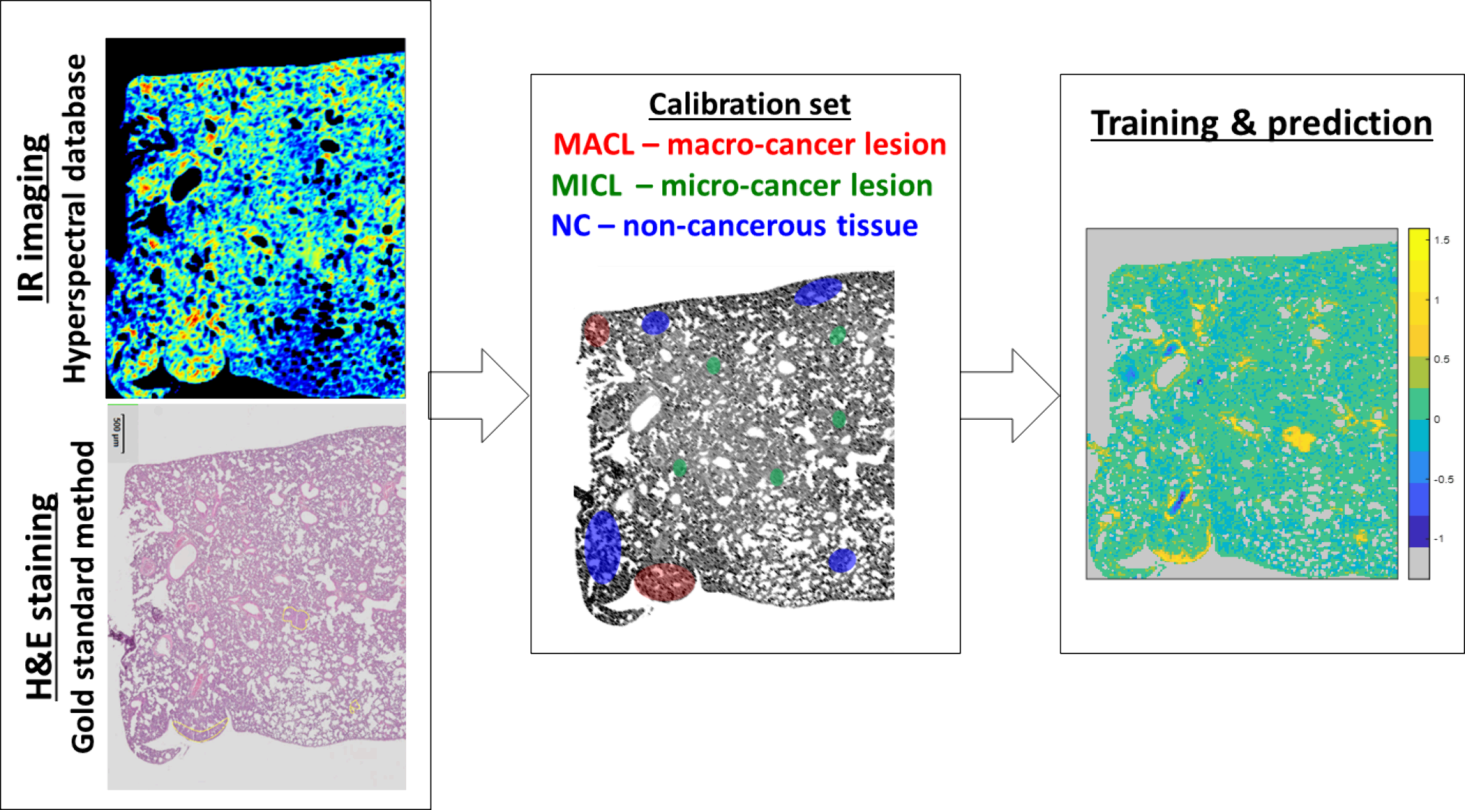
Overview of the workflow used in the design and validation of FT-IR digital image analysis and cancer lesion prediction.

**Table 1:** Discriminant Analysis and Feature Selection in Hyperspectral Imaging Using CORRS-CV^a^

**data sets**								
			number of pixels					
		**imaged area [mm** **^2^** **]**	total	not assigned	MICL	MACL	NC	control
lungs with cancer cells	section I	1.49	49,152	47,013	54		2085	
	section II	1.115	36,864	17,532	12	8	880	
	section III	0.929	30,720	28,618		95	2007	
	section IV	1.084	35,840	33,761	64	468	1547	
	section V	0.929	30,720	28,918	16	38	1748	
	section VI	0.929	30,720	29,269	42		1409	
	section VII	0.867	28,672	26,975	102		1594	
	section VIII	1.487	49,152	47,121	25	202	1904	
	section IX	0.929	30,720	28,179		218	2323	
healthy control	section X	0.992	32,768	32,194				574
	section XI	1.238	40,960	40,132				828
	section XII	1.734	57,344	55,588				1756
	section XIII	1.487	49,152	48,324				828
	section XIV	1.487	49,152	48,524				628
Summary		16.697	551,936	512,148	315	1029	13,233	4419

a MICL: microcancer lesion; MACL: macrocancer lesion; NC: noncancerous.

In the absence of a fully independent external cohort, the classification performance was assessed using three cross-validation (CV) strategies: repeated random *k*-fold CV, image-based CV, and CORRS-CV.^27,31^ In random *k*-fold CV (*k* = 10), the multi-image data set was randomly split into 10 folds. Nine folds were used to train a discriminant model, which was then used to predict the remaining fold. The process was repeated *k* times until each fold had been predicted once, and the overall model performance was calculated as the average across the 10 submodels. To account for variability introduced by the random partitioning, the procedure was repeated five times, and the mean prediction error was reported. However, *k*-fold CV leads to a loss of spatial information, allowing spatially proximate pixels to be included in both the training and test sets, resulting in a situation resembling the replicate trap and providing overly optimistic classification accuracy estimates. To mitigate this, an image-based CV procedure was implemented, with each fold corresponding to an entire image. This approach minimizes the risk of information leakage between the training and test sets, but its applicability can be limited when the number of available images is small, as only a few folds can be generated and fold-to-fold variability may increase. Finally, CORRS-CV was applied. In this strategy, training and test subsets are defined by constrained random sampling of pixels without replacement, ensuring that both (i) the distance among training pixels and (ii) the distance between training and test pixels exceed a user-defined threshold (∂). This spatial constraint reduces overestimation of performance while enabling the use of all available images for model development. Thus, CORRS-CV provides a practical compromise: it preserves the advantages of pixel-level validation across the entire data set while imposing spatial constraints that reduce the optimism of standard *k*-fold CV. CORRS-CV was applied using the following parameters: ∂ = 5; repeated (*n* = 5) random 10-fold CV for the CV and optimization of inner PLS models; at least 99% of labeled pixels included at least one time in a training set, once in a test set, and once in the prediction set; number of pixels in each training set = 200; latent variable selection based on the minimum mean CV classification error. The random *k*-fold CV results are reported for comparison only; model robustness and conclusions are primarily derived from image-based CV and CORRS-CV, which explicitly prevent sample-level and spatial data leakage.

## Results and Discussion

### Automated Recognition of Neoplastic Lesions Using FT-IR Hyperspectral Imaging

The lungs constitute a large organ characterized by an extensive network of blood vessels, which render their structure particularly delicate and susceptible to pathological alterations.^32^ Due to the presence of numerous major and minor vessels, as well as bronchioles and airways, malignant transformation can occur not only as a primary neoplasm but also as secondary tumors originating from distant organs.^33^ The coexistence of sizable and small blood vessels, bronchioles, and conducting airways provides a microenvironment in which malignant transformations may occur, both as primary neoplasms and as metastatic foci originating from distant sites. In the framework of histopathological evaluation, the initial diagnostic procedure involves biopsy examination followed by H&E staining. This classical approach permits the visualization of cell nuclei (stained dark blue) and cytoplasm (stained pink) (Figure 2A–D).

**Figure 2:**
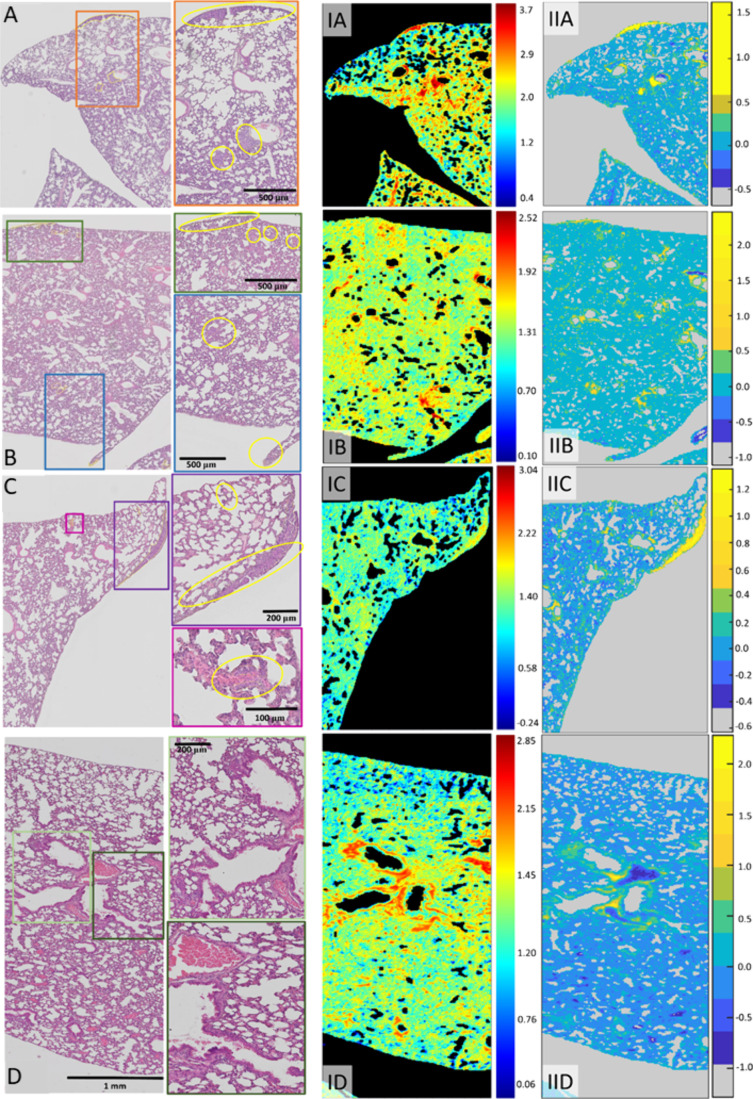
Lung tissue cross sections stained with H&E with marked regions of micro- and macrocancer lesions (yellow) (A–C) and healthy control (D) accompanied by magnified microphotographs of the indicated areas. Corresponding protein distribution maps obtained from amide I band integration in FT-IR spectra (IA–ID), along with prediction maps generated by AI-assisted analysis showing localization of cancerous regions (IIA–IID, yellow).

Upon dissemination, neoplastic cells display a broad spectrum of infiltration strategies.^34,35^ They may adhere to and proliferate within the pleura, which is observed as thickening of the compact tissue located at the periphery of lung cross sections (Figure 2A–C, orange, green, and violet frames, respectively). Furthermore, malignant cells can penetrate vascular and bronchial walls (Figure 2A, lower orange frame; Figure 2B, purple frame) or migrate through the smallest capillaries (∼15 μm in diameter), subsequently establishing colonies within the parenchyma (Figure 2B, blue frame). The ability to recognize such a wide heterogeneity of tumor localizations within a single histological section was only made possible with an animal model. In this experimental setting, 4T1 breast carcinoma cells were directly inoculated into the mammary gland, enabling longitudinal monitoring of tumor progression and its metastatic dissemination to pulmonary tissues. Macroscopically visible lesions are easily identifiable as well-delineated regions of tissue thickening, resulting from the accumulation of neoplastic cells (exemplary size: 0.2 mm^2^). However, numerous smaller foci are also present, namely, microscopic lesions of average size ca. 0.000733 mm^2^, which are considerably more challenging to identify and can be readily overlooked during conventional microscopic examination. Detecting individual malignant cells with an approximate diameter of 13 μm across a full lung cross section (∼20 mm^2^) is an especially laborious and time-consuming task. However, they are recognized by unique clustered FT-IR features as we have shown previously.^7,13,36,37^ Despite these obstacles, H&E staining persists as the gold standard methodology, providing a robust and reliable reference framework, albeit requiring several days of meticulous manual analysis.

An attractive alternative to support such a comprehensive examination was the utilization of FT-IR spectroscopy. Using the FPA detector with a 128 × 128-pixel array (projected pixel size of 5.5 μm × 5.5 μm), it was possible to image a region of interest of approximately 0.5 mm^2^ within 15 min. This measurement yields 16,384 pixel-dependent spectra acquired at a spectral resolution of 4 cm^–1^, with each spectrum providing biochemical information from an area of 30.25 μm^2^. Such an approach enables the independent construction of a hyperspectral database and facilitates the correlation of morphological features with their underlying biochemical modifications. In this way, IR spectroscopy imaging not only enables the detection of neoplastic lesions but also distinguishes them by their spatial localization, providing chemical contrast beyond conventional histopathology.^29^ Based on such data sets, hyperspectral images can be reconstructed to represent the analyzed tissue (Figure 2IA–ID) and visualized according to the spatial distribution of specific molecular components, in this case the distribution of proteins through the integrated amide I band. As mentioned above, large cancer lesions appear as dense cellular aggregates, visualized in IR image as pixels with the highest intensity associated with regions of elevated protein concentration. Interestingly, a similar pattern was also observed in healthy lung cross sections, where strong protein signals were primarily associated with vascular walls (Figure 2ID).

Access to such a comprehensive database, comprising both IR-imaged cross sections and their corresponding H&E-stained microphotography enabled precise annotation of areas occupied by micro- and macrocancer lesions, as well as noncancerous tissue and healthy controls (Table 1). This was applied to construct the calibration data set used in subsequent predictive modeling, where cancerous classes were defined as micro- (MICL, 315 pixels) and macrocancer lesions (MACL, 1029 pixels). In addition, 13,233 pixels from tumor-bearing lung sections, selected from noncancerous regions, and 4419 pixels from healthy control sections were included. The predictive images accurately delineate areas occupied by cancerous lesions (Figure 2IIA–IIC, yellow spots), consistent with histopathological annotations (Figure 2A–C). Notably, the model also identified additional regions with spectral features like those of cancerous tissues, suggesting potential tumor presence beyond visibly affected areas. Pixel-wise spectral assignment thus enabled precise imaging of cancer-occupied regions. As mentioned above, high-intensity pixels indicative of elevated protein content often colocalize with neoplastic lesions; however, they may also reflect protein accumulation in vessel walls, as observed in control samples (Figure 2IID).

### Assessment of the Predictive Performance of PLS-DA Models for Cancer Classification

In the comparative evaluation of three discriminant models for differentiating NC from MICL and MACL cancer lesions, all approaches demonstrated high predictive performance, though with notable differences in specificity and overall accuracy (Table 2).

**Table 2:** Discriminant Analysis Strategies in Hyperspectral Imaging

discriminant analysis of noncancerous (NC), macro- (MACL), and microcancer (MICL) lesion						
	5-iteration 10-fold random CV		image-based CV LVs = 4		CORRS-CV δ = 5	
	confusion table					
	actual class (CV)		actual class (CV)		actual class (test set)	
	MACL, MICL	NC	MACL, MICL	NC	MACL, MICL	NC
predicted as MACL, MICL	1179	404	1173	474	1176	247
predicted as NC	165	12,829	171	12,759	168	12,986
	AUROCcv = 0.96		AUROCcv = 0.96		AUROC test = 0.9398 ± 0.01 (as the mean of AUROCs of the set of iterations)	
sensitivity	87.72 (85.85–89.43)%		87.28 (85.38–89.01)%		87.50 (85.61–89.22)%	
specificity	96.95 (96.64–97.23)%		96.42 (96.09–96.73)%		98.13 (97.89–98.36)%	
positive likelihood ratio	28.73 (26.05–31.69)		24.37 (22.25–26.68)		46.88 (41.36–53.13)	
negative likelihood ratio	0.13 (0.11–0.15)		0.13 (0.11–0.15)		0.13 (0.11–0.15)	
accuracy	96.10 (95.77–96.41)%		95.58 (95.23–95.90)%		97.15 (96.87–97.42)%	

The 5-iteration 10-fold random CV model achieved an AUROC of 0.96, with a sensitivity of 87.72%, a specificity of 96.95%, and an overall accuracy of 96.10%. The positive likelihood ratio (LR^+^) reached 28.73, indicating strong discriminative ability, while the negative likelihood ratio (LR^–^) was 0.13, reflecting that a negative test result substantially decreases the probability of cancer presence. The image-based CV model with four latent variables (LVs) performed comparably in terms of AUROC (0.96); however, it showed slightly lower specificity and accuracy (96.42% and 95.58%, respectively). It also yielded a reduced LR^+^ of 24.37, reflecting a weaker separation between cancerous and noncancerous classes compared to the random CV model. The CORRS-CV δ = 5 model provided the most robust performance, with an AUROC of 0.94 across iterations, a sensitivity of 87.50%, and the highest specificity of 98.13% among the tested approaches. Its LR^+^ was markedly superior at 46.88, underscoring its ability to strongly confirm cancerous lesions when it was predicted as positive. Importantly, the accuracy of CORRS-CV (97.15%) surpassed both alternative models. This improvement can be attributed to the better handling of interimage correlations. By enforcing a stricter δ threshold, CORRS-CV reduced false-positive classifications in NC cases, thereby enhancing specificity without sacrificing sensitivity. Taken together, while all models achieved a balanced sensitivity of ∼87%, the CORRS-CV approach demonstrated the best trade-off between sensitivity and specificity, yielding the highest accuracy and strongest likelihood ratios, and thus represents the most reliable strategy for discriminating between NC, MICL, and MACL cases.

Figure 3A–C presents the discriminant analysis comparing noncancerous (NC) tissues with macrocancer lesions (MACL).

**Figure 3:**
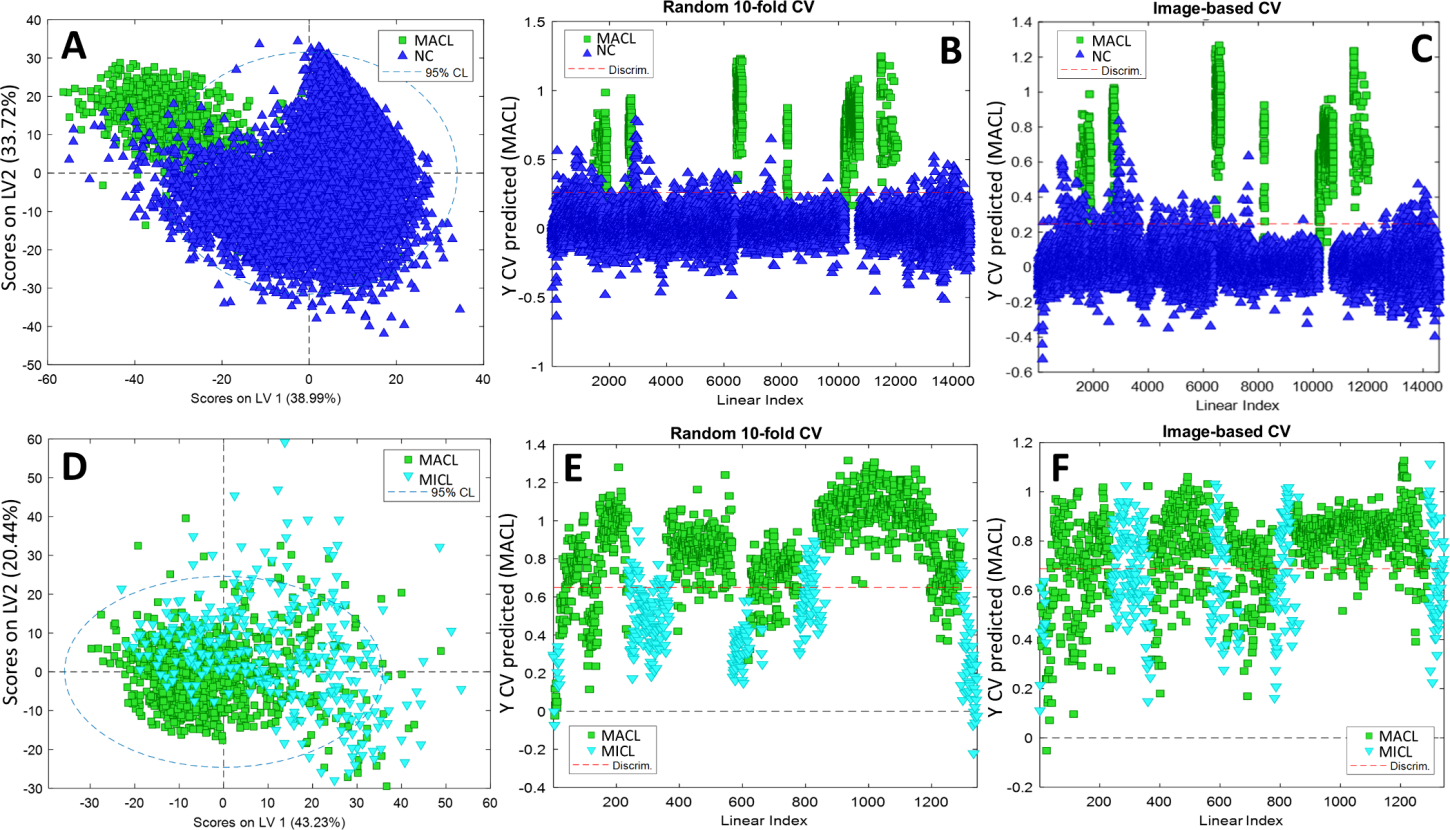
(A–C) Discriminant analysis of noncancerous vs macrocancer lesions and (D–F) and macro- vs microcancer lesions.

The score plots indicated a tight clustering of MACL pixels and a higher within-group variability in NC pixels (Figure 3A). Besides the limited overlap, the applied model successfully captured the major biochemical differences between normal and malignant tissues. Results from random 10-fold (AUROCcv = 0.9886) and image-based CV (AUROCcv = 0.9817) (Figure 3B,C) confirmed the high discriminatory power of the PLS-DA model. Most NC spectra were correctly classified, while MACL spectra formed compact and distinct clusters, with only a small proportion of misclassifications. In contrast, discrimination between cancer lesion sizes (MICL vs MACL) was more challenging. The score plot revealed partial overlap between the two subtypes, consistent with their shared pathological features (Figure 3D). Nonetheless, the classification plots demonstrated statistically significant discrimination, suggesting that FT-IR spectroscopy can detect subtle molecular differences underlying lesion size (Figure 3E,F). These findings indicated that FT-IR-based SHI, combined with multivariate discriminant analysis, could not only distinguish cancerous from noncancerous tissues but also stratify tumors according to the progression stage. Moreover, the choice of cross-validation strategy influenced the apparent discrimination between groups. While random *k*-fold CV yielded higher classification efficiency (Ecv = 0.93), image-based CV provided a more conservative estimate (*E* = 0.66). This difference highlighted that pixel distribution across images can affect model generalizability estimates as limited image numbers, unbalanced class representation, and interimage spectral variation can all bias performance estimates. Such effects may be partly circumvented by optimized spectral preprocessing or more advanced CV approaches, underscoring the need for careful selection of CV strategies in SHI studies.

### Misclassification of Vascular Structures in Automated Cancer Detection

During automated classification of neoplastic lesions in the lung, the algorithm occasionally assigned malignant cells to vessel walls not affected by cancer (Figure 4A–C). H&E staining of a representative lung cross section revealed multiple morphological structures, including macro- and microcancer lesions (MACL and MICL, Figure 4A; red and green arrows, respectively), bronchiolar walls containing cancer infiltration (Figure 4A; blue arrows), and vessel walls either classified (orange arrows) or not classified (gray arrows) as harboring malignant cells (Figure 4A). The two prediction images reproduced both MACL and MICL and additionally highlighted additional malignant foci within the parenchyma (Figure 4B,C; yellow areas). However, when the calibration set included both MICL and MACL pixels (Figure 4B), strong cancer predictions also appeared in the vessel walls (Figure 4A; orange arrows). In contrast, when only MACL pixels were used against the noncancer (NC) class, this artifact was less pronounced (Figure 4C).

**Figure 4:**
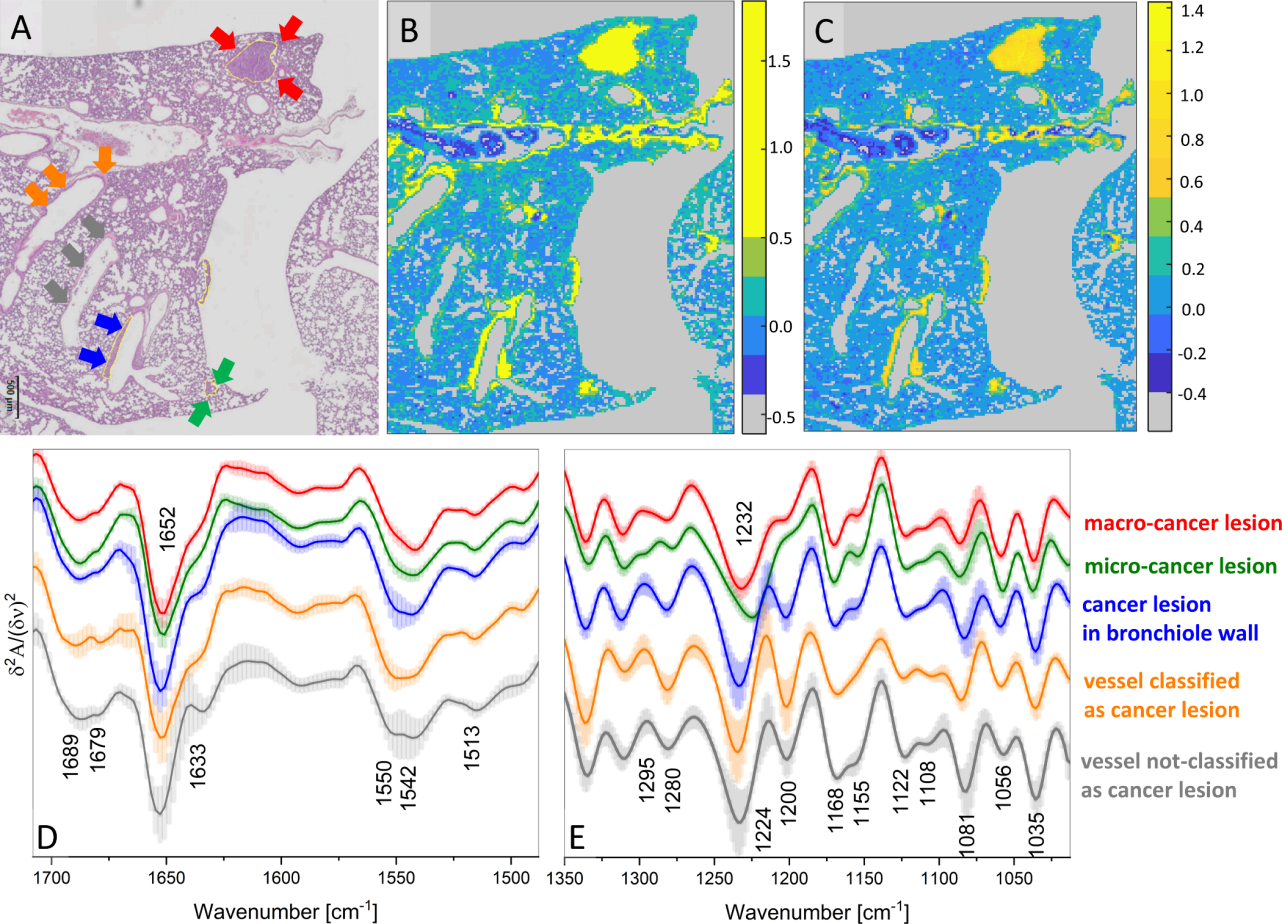
(A) H&E staining with marked macro- and microcancer lesions in the parenchyma (red and green arrows, respectively), cancer lesion in the bronchiole wall (blue arrows), vessel walls classified and not classified as cancerous (orange and gray arrows, respectively) in (B) and (C). (B) Prediction image for the macro- and microcancer lesions vs noncancerous models. (C) Prediction image for the macrocancer lesion vs noncancerous models. (D, E) Second-derivative FT-IR spectra averaged from 10 pixels within labeled tissue structures in (A) (shading denotes standard deviation).

To assess this effect, spectra were extracted from each of the identified morphological components (10 spectra per structure) and compared as second derivatives (Figure 4D,E). All cancer-assigned cases exhibited a low-intensity shoulder of the β-sheet amide I band at 1633 cm^–1^, whereas only the lesions within the bronchial walls showed more prominent collagen-associated features in the 1200–1300 cm^–1^ region, as in the noncancer vessel wall.^7^ Comparison of the vessel spectra revealed distinct signatures. The noncancer classified vessel (gray trace) also exhibited strong collagen-associated bands at 1081 cm^–1^, while the misclassified vessels (orange trace) were characterized by a single prominent 1168 cm^–1^ band. Although nonclassified vessels shared several ECM remodeling features with malignant lesions (e.g., 1168 and 1155 cm^–1^), these bands did not dominate the PLS-DA prediction model.^38^ Given that lungs are predominantly composed of vessels and parenchyma, spectral contributions from vascular structures are expected, especially due to the abundance of collagen. While less pronounced β-sheet-related amide I features (∼1633 cm^–1^) were consistently present in all cancer-assigned spectra, collagen-dominated bands (1200–1300 and 1081 cm^–1^) contributed to false-positive predictions when micro- and macrolesion spectra were jointly included in the calibration set. This biochemical overlap likely explains the misclassification of some vessel walls as a neoplastic tissue; however, they can be easily verified by a histopathologist.

## Conclusions

This study demonstrates the feasibility and utility of AI-assisted FT-IR hyperspectral imaging for automated histopathology screening of lung cancer lesions. By integrating chemometric models with annotated spectral data sets, we achieved reliable discrimination between noncancerous tissues and micro- and macrolesions. Among the tested approaches, the CORRS-CV strategy provided the most accurate and specific classification, underscoring the importance of appropriate cross-validation design in hyperspectral studies.

Our findings further highlight the potential of FT-IR spectroscopy and its imaging modality to detect subtle biochemical differences associated with tumor progression, enabling recognition of microscopic lesions that may be overlooked by conventional histopathology. At the same time, occasional misclassification of vascular structures emphasizes the need for refined preprocessing and model optimization to mitigate spectral overlap with collagen-rich tissues.

Overall, this work establishes FT-IR hyperspectral imaging, enhanced by AI-driven chemometrics, as a powerful complementary tool to conventional staining methods. Its ability to deliver rapid, label-free, and spatially resolved analysis positions it as a promising strategy for improving diagnostic accuracy, accelerating screening workflows, and ultimately supporting early cancer detection and patient stratification.

## Data Availability

The script is available at Zenodo (DOI: 10.5281/zenodo.1740328).
